# The prevalence and clinicopathological features of programmed death-ligand 1 (PD-L1) expression: a pooled analysis of literatures

**DOI:** 10.18632/oncotarget.7590

**Published:** 2016-02-22

**Authors:** Ziying Lin, Yutong Xu, Yaxiong Zhang, Qihua He, Jianrong Zhang, Jianxing He, Wenhua Liang

**Affiliations:** ^1^ Department of Thoracic Surgery and Oncology, The First Affiliated Hospital of Guangzhou Medical University, Guangzhou, China; ^2^ Guangzhou Institute of Respiratory Disease & China State Key Laboratory of Respiratory Disease & National Clinical Research Center for Respiratory Disease, Guangzhou, China; ^3^ Zhongshan School of Medicine, Sun Yat-sen University, Guangzhou, China; ^4^ Department of Medical Oncology of Sun Yat-sen University Cancer Center, Guangzhou, China

**Keywords:** programmed death-ligand 1, clinicopathological features, cancer, meta-analysis

## Abstract

**Background & Aims:**

Programmed death-ligand 1 (PD-L1) has been recognized as a critical and promising target in therapies that direct immune escape of cancers. However, its association with aggressive clinicopathological features in solid tumors remains unclear. We investigated this question by synthesizing published articles.

**Methods:**

Electronic databases were searched for relevant studies. Outcomes of interest included age, gender, tumor size, tumor size, lymph node metastasis and tumor cell differentiation.

**Results:**

A total of 61 studies involving 17 types of malignancies were included. The overall expression rate of PD-L1 was 44.5% (95% CI, 37.5% to 51.6 %). Patients with regional lymph node metastases (OR 1.38; P < 0.01), large size tumor (OR 1.89; P < 0.01) or poor differentiated tumors (OR 1.71; P < 0.01) were associated with higher PD-L1 expression rate. However, no significant association was observed between young and elder patients (OR 1.04; P = 0.58), or male and female patients (OR 1.13; P = 0.06). A numerically higher PD-L1 expression rate was detected in polyclonal antibodies (57.2%) than monoclonal antibodies (39.6%). In addition, the PD-L1 expression rate reported by studies from Asian areas (52.3%) was numerically higher than those from non-Asian areas, namely Caucasians (32.7%).

**Conclusions:**

This meta-analysis indicated that patients with larger tumors, regional lymph node metastases, or poor-differentiated tumors were associated with a higher PD-L1 expression rate; in addition the expression rate of PD-L1 in Asians might be higher than that of Caucasians. This information might be useful in screening candidates for relevant tests and treatments.

## INTRODUCTION

Programmed cell death ligand 1 (PD-L1), also known as B7 homolog 1 (B7H1), plays a critical role in T cells co-inhibition and exhaustion via the binding with programmed death 1 (PD-1) receptor expressed on activated T cells[1, 2]. Recently, it's reported that PD-L1 expressed on tumor cells can promote anergy or apoptosis of tumor antigen-specific T-cells, resulting in enhanced tumor cell growth and tumor immune evasion [2, 3].

PD-L1 expression has been observed in various solid tumors, including carcinomas of the esophagus, gastrointestinal tract, pancreas, breast, lung, and kidney [4–8]. Inhibition of the PD-1/PD-L1 axis may become a promising approach to restore host immunity against these tumors [9, 10].

Recent clinical trials show that blockage of PD-1/PD-L1 pathway with anti-PD-1 or anti-PD-L1 antibodies can produce durable remission in several different malignancies including, but not limited to, melanoma, lung, renal and bladder cancer[11–14]. Although the response is remarkable, the response rate is quite low, which means only a small subset of patients benefit from PD-1/PD-L1-directed immunotherapy [15, 16]. Further research has demonstrated that clinical response was closely associated with PD-L1 expression[17]. However, screening the target population is difficult due to a lack of uniform standard in PD-L1 detection [18]. Therefore, determining the subset of patients with positively expressed PD-L1 in tumor cells is of great clinical significance, particularly in light of the autoimmune toxicity of immunotherapy.

A number of recent studies have reported on the clinicopathologic features of PD-L1 positive cancer. However the results of these studies were not consistent and sample sizes were relatively modest. Since PD-L1/PD-1 is a common pathway that functions in a wide spectrum of cancers, we performed a meta-analysis by incorporating all the available evidence to evaluate the association between clincopathological features and PD-L1 expression, aiming to identify those with high PD-L1 expression that may benefit from anti-PD-L1 therapy.

## RESULTS

### Search results

**Figure 1:**
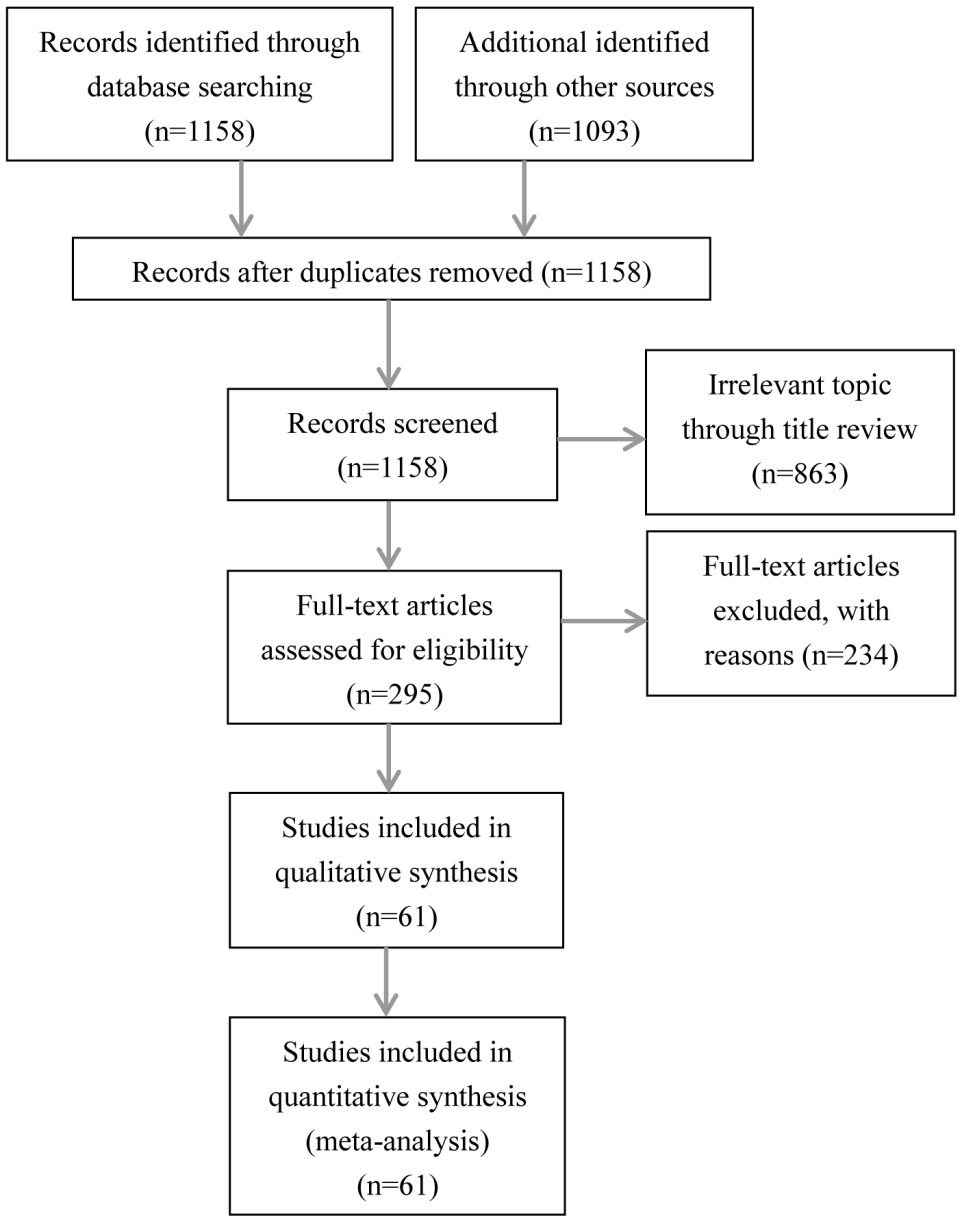
Flow chart of study selection

Our initial search yielded 1158 potential literature citations. Of these, 863 were excluded after scanning titles and abstracts, leaving 295 citations for full-text assessment. Of the 295, 61 studies met the inclusion criteria and were used for this meta-analysis. The remaining studies were excluded largely because of the following reasons: (I) reported data regarding clinicopathological features was not stratified by PD-L1 status, (II) the data was reported in an unsuitable format and therefore unable to be compared to the others studies, or (III) they included data that overlapped with other studies. (Figure 1)

### Characteristics of the studies

Baseline characteristics of the studies included are shown in Table 1. The 61 included studies covered 17 types of epithelial-originated malignancies, including adrenocortical carcinoma (AC) breast cancer (BC), cervical carcinoma (CC), clear cell renal cell carcinoma (CRCC), non-clear cell renal cell carcinoma (NCRCC), colorectal cancer (CRC), esophageal cancer (EC), gastric carcinoma (GC), hepatocellular carcinoma (HCC), head and neck cancer (HNC), non-small cell lung cancer (NSCLC), small cell lung cancer (SCLC), pancreatic cancer (PC), plural mesothelium, skin cancer (SK), thymic carcinoma (TC) and urothelial cell carcinoma (UCC). The total number of patients of epithelial-originated cancer was 9212, including 4068 cases of Asian origin. The overall expression rate of PD-L1 was 44.5% (95% CI, 37.5% to 51.6 %). Mouse-originated monoclonal antibody accounted for the vast majority in terms of primary anti-PD-L1/PD-1 antibody. In the subgroup analysis of PD-L1 expression rate (more details are in Table 2), we found a numerically higher PD-L1 expression rate when using polyclonal antibodies 57.2% (95% CI 49.8% to 64.6%) than monoclonal antibodies 39.6% (95% CI 32.8% to 46.3%). In addition, the PD-L1 expression rate reported by studies from Asian areas was numerically higher than those from non-Asian areas, namely Caucasians 52.3% (95% CI 46.9% to 57.6%) and 32.7% (95% CI 24.3% to 41.1%) respectively. Not all studies reported on all variables examined in the meta-analysis; therefore, only studies that reported the variable of interest were analyzed for PD-L1 association with that variable. Funnel plots with the Begg's tests of different clinicopathological parameters were shown as Supplementary Figure S1.

**Table 1:** Characteristics of included studies for meta-analysis

Lead author (y)	Tumor type	Patients' origin	IHC evaluation^a^	Cut-off value for PD-L1 positive	Antibody				PD-L1 positive/total	PD-L1 positive (%)
					Company	Source	Type	Catalog		
Fay(2015)	AC	Non-Asia	Percentage	≥5%	NA	NA	MAB	NA	3/28	10.71
GhebehH(2006)	BC	Asia	Percentage	≥5%	eBioscience, USA	Mouse	MAB	clone MIH1	13/44	29.55
Ghebeh H(2008)	BC	Asia	Percentage	≥5%	eBioscience, USA	Mouse	MAB	clone MIH1	19/62	30.65
Mittendorf EA(2014)	BC	Non-Asia	Percentage	≥5%	Lieping Chen, Yale University	Mouse	MAB	clone 5H1	20/105	19.04
Muenst S(2014)	BC	Non-Asia	H-score	≥100	Abcam, UK	Rabbit	PAB	NA	152/650	23.38
Karim R(2009)	CC	Non-Asia	Percentage	>0	Lieping Chen, Yale University	Mouse	MAB	clone 5H1	22/115	19.13
Droeser RA(2013)	CRC	Non-Asia	H-score	≥200	MBL, USA	Mouse	MAB	clone 27A2	669/1420	47.11
Hua D(2012)	CRC	Asia	H-score	≥200	NA	NA	NA	NA	15/33	45.45
Liang M(2014)	CRC	Asia	H-score	≥20	Santa Cruz Biotechnology, USA	Rabbit	PAB	NA	102/185	55.14
Shi SJ(2013)	CRC	Asia	H-score	≥200	Abcam, UK	Rabbit	PAB	ab58810	64/143	44.76
Zhang MY(2012)	CRC	Asia	Percentage	≥10%	NA	NA	NA	NA	26/57	45.61
Zhao LW(2014)	CRC	Asia	H-score	≥20	eBioscience, USA	Mouse	MAB	clone MIH1	27/56	48.21
Krambeck AE(2007)	CRCC	Non-Asia	Percentage	≥5%	Lieping Chen, Yale University	Mouse	MAB	clone 5H1	70/298	23.49
Thompson RH(2005)	CRCC	Non-Asia	Percentage	≥5%	Lieping Chen, Yale University	Mouse	MAB	clone 5H1	103/196	52.55
Thompson RH(2006)	CRCC	Non-Asia	Percentage	≥5%	Lieping Chen, Yale University	Mouse	MAB	clone 5H1	73/306	23.86
Thompson RH(2007)	CRCC	Non-Asia	Percentage	≥10%	Lieping Chen, Yale University	Mouse	MAB	clone 5H1	142/267	53.18
Loos M(2011)	EC	Non-Asia	H-score	≥66	Abcam, UK	Rabbit	PAB	NA	37/101	36.63
Ohigashi Y(2005)	EC	Asia	Percentage	≥10%	eBioscience, USA	Mouse	MAB	clone MIH1	18/41	43.9
Shohei Eto(2015)	GC	Asia	H-score	≥51	Abcam, Cambridge, UK	Rabbit	MAB	ab174838	28/105	26.7
Geng Y(2014)	GC	Asia	H-score	≥22	Novus, USA	Mouse	MAB	clone 2H11	65/100	65
Hou J(2014)	GC	Asia	Percentage	≥10%	Abcam, UK	Rabbit	PAB	NA	70/111	63.06
Wu C(2006)	GC	Asia	NA	NA	Novus, USA	Mouse	MAB	clone 2H11	43/102	42.16
Gao Q(2009)	HCC	Asia	H-score	≥75th percentile	eBioscience, USA	Mouse	MAB	clone MIH1	60/240	25
Wang BJ(2011)	HCC	Asia	H-score	≥10	eBioscience, USA	Mouse	MAB	clone MIH1	24/26	92.31
Cho YA(2011)	HNC	Asia	H-score	≥Median	Abcam, UK	Rabbit	PAB	ab82059	26/45	57.78
Oliveira-Costa JP(2015)	HNC	Non-Asia	NA	NA	NA	NA	NA	NA	47/96	48.96
Ukpo OC(2013)	HNC	Asia	Percentage	≥5%	NA	NA	MAB	clone A3	84/181	46.41
Zhang F(2008)	HNC	Asia	Percentage	≥10%	Boster, China	Rabbit	PAB	BA2495	40/59	67.8
Choueiri(2014)	NCRCC	Non-Asia	Percentage	≥5%	Dana-Farber Cancer Institute, Boston, MA	mouse	MAB	405.9A11	11/101	10.89
Azuma K(2014)	NSCLC	Asia	H-score	>30	Lifespan Biosciences, WA	Rabbit	PAB	NA	82/164	50
Boland JM(2013)	NSCLC	Non-Asia	Percentage	≥5%	Laboratory developed	NA	NA	clone 5H1	42/214	19.63
Chen YB(2012)	NSCLC	Asia	H-score	≥3	Abcam, HK	Rabbit	PAB	clone 236A/E7	69/120	57.5
Chen YY(2013)	NSCLC	Asia	H-score	>9	Abcam, HK	Rabbit	PAB	NA	136/208	65.38
Cooper, W. A(2015)	NSCLC	Non-Asia	percentage	≥50%	Merck	Mouse	MAB	clone22C3	628/678	92.6
D'Incecco(2015)	NSCLC	Non-Asia	H-score	≥10	Abcam, UK	Rabbit	PAB	ab58810	68/123	55.3
Jiang(2015)	NSCLC	Asia	Percentage	≥5%	Abcam, UK	Rabbit	PAB	NA	50/79	63.29
Kim(2015)	NSCLC	Asia	H-score	≥30	Cell Signaling Technology, Danvers, MA, USA	NA	MAB	E1L3N	89/331	26.89
Mu CY(2011)	NSCLC	Asia	H-score	≥Median value	NA	NA	MAB	NA	58/109	53.21
Velcheti V(2014)	NSCLC (Yale U. cohort)	Non-Asia	DIA	NA	Lieping Chen, Yale University	Mouse	MAB	clone 5H1	56/155	36.13
Velcheti V(2014)a	NSCLC(Greek cohort)	Non-Asia	DIA	NA	Lieping Chen, Yale University	Mouse	MAB	clone 5H1	75/303	24.75
Yang CY(2014)	NSCLC	Asia	Percentage	≥5%	Proteintech Group Inc., USA	Rabbit	PAB	17952-1-AP	65/163	39.88
Yih-Leong Chang(2015)	NSCLC	Asia	NA	NA	Proteintech Group Inc	Rabbit	PAB	NA	50/66	75.76
Zhang Y(2014)	NSCLC	Asia	H-score	≥60	Sigma-Aldrich, USA	Rabbit	PAB	SAB2900365	70/143	48.95
Ishii(2015)	SCLC	Asia	Percentage	≥5%	Abcam, UK	Rabbit	PAB	NA	73/102	71.56
Chen XL(2009)	PC	Asia	Percentage	≥10%	Novus, USA	Mouse	MAB	clone 2H11	18/40	45
Geng L(2008)	PC	Asia	Percentage	≥10%	R&D systems	Mouse	MAB	Clone130002	23/40	57.5
Nomi T(2007)	PC	Asia	Percentage	≥10%	eBioscience, USA	Mouse	MAB	clone MIH1	20/51	39.22
Wang L(2010)	PC	Asia	Percentage	≥5%	eBioscience, USA	Mouse	MAB	clone MIH1	40/81	49.38
SusanaCedrés(2015)	Plural mesothelium	Non-Asia	Percentage	≥1%	Cell Signaling Technology, Danvers, MA, USA	Rabbit	MAB	cloneE1L3N	16/77	20.78
Gadiot J(2011)	SC (Melanoma)	Non-Asia	Percentage	≥1%	R&D Systems, USA	Mouse	MAB	156-B7-100	16/63	25.4
Hino R(2010)	SC (Melanoma)	Asia	DIA	RD^b^≥90	MBL, Japan	Mouse	MAB	clone 27A2	34/59	57.63
Oba J(2014)	SC (Melanoma)	Asia	Percentage	≥5%	eBioscience, USA	Mouse	MAB	clone MIH1	36/77	46.75
Taube JM(2012)	SC (Melanoma)	Non-Asia	Percentage	≥5%	Lieping Chen, Yale University	Mouse	MAB	clone 5H1	57/150	38
Lipson EJ(2013)	SC (Merkel cell carcinoma)	Non-Asia	Percentage	≥5%	Lieping Chen, Yale University	Mouse	MAB	clone 5H1	24/49	48.98
Katsuya (2015)	TC	Asia	H-score	3	Cell Signaling Technology, Danvers, MA, USA	Rabbit	MAB	NA	48/139	34.53
Boorjian SA(2008)	UCC	Non-Asia	Percentage	≥5%	Lieping Chen, Yale University	Mouse	MAB	clone 5H1	39/314	12.42
Faraj (2015)	UCC	Non-Asia	Percentage	≥5%	NA	Mouse	MAB	clone 5H1	10/56	17.86
Inman BA(2007)	UCC	Non-Asia	Percentage	≥1%	Lieping Chen, Yale University	Mouse	MAB	clone 5H1	77/280	27.5
Nakanishi J(2006)	UCC	Asia	Percentage	≥2.1%	eBioscience, USA	Mouse	MAB	clone MIH1	46/65	70.77
Wang Y(2009)	UCC	Asia	Percentage	>10%	Santa Cruz Biotechnology, USA	Rabbit	PAB	NA	36/50	72
Wang YH(2014)	UCC	Asia	Percentage	≥10%	Boster, China	Rabbit	PAB	BA2495	43/60	71.67

a H-score = SI (staining intensity) × PP (percentage of positive cells). SI was determined as: 0, negative; 1, weak; 2, moderate; and 3, strong. PP was defined as: 0, negative; 1-100, 1-100% positive cells

b Digitized specimens were exported to JPG files by using NDP View software (Hamamatsu Photonics). Three different areas from the tumor cell cytoplasm were selected and expressed as Red channel histograms. Histograms revealed 255 different shades from pitch black (0) to pure white (255), and a number represented the level of brightness of each color. RD (red density) values represent the mean of each color

Abbreviations: AC = adrenocortical carcinoma; BC = breast cancer; CC = cervical carcinoma; CRCC = clear cell renal cell carcinoma; NCRCC = non-CRCC; CRC = colorectal cancer; EC = esophageal cancer; GC = gastric carcinoma; HCC = hepatocellular carcinoma; HNC =head and neck cancer; SCLC = small cell lung cancer; NSCLC = non-SCLC; PC = pancreatic cancer; SC=skin cancer; TC = thymic carcinoma; UCC = urothelial cell carcinoma; DIA = digital image analysis; IHC = immunohistochemistry; MAB = monoclonal antibody; NA = not available; PAB= polyclonal antibody; PD-L1 = Programmed cell death ligand 1.

**Table 2:** Summary of PD-L1 expression rate in subgroup analysis

Subgroup	*No*. of studies	PD-L1 expresson rate (95% CI) / %	Subgroup	*No*. of studies	PD-L1 expression rate (95% CI) / %
**Tumor type**			**Patients' origin**		
adrenocortical carcinoma	1	10.7 (−0. 7-22.1)	Asian	37	52.3 (46.9-57.6)
Breast cancer	4	23.7 (19.9-27.4)	Non-Asian	24	32.7 (24.3- 41.1)
Cervical carcinoma	1	19.1 (11.9-26.3)	**Antibody-Type**		
Clear cell renal cell carcinoma	4	38.1 (22.0-54.2)	Monoclonal antibody	39	39.6 (32.8-46.3)
Non-clear cell renal cell carcinoma	1	10.9 (4.8-17.0)	Polyclonal antibody	18	57.2 (49.8-64.6)
Colorectal cancer	6	47.7 (45.4-49.9)	**IHC evaluation**		
Esophageal cancer	2	38.6 (30.7-46.6)	Percentage	35	42.8 (31.5-54.2)
Gastric carcinoma	4	49.2 (30.8-67.6)	H-score	20	47.7 (40.5-54.9)
Hepatocellular carcinoma	2	58.5 (−7.5-124.5)	DIA	3	24.9 (20.4-29.3)
Small cell lung cancer	1	71.6 (62.8-80.3)	NA	3	55.6 (35.7-75.4)
Non-small cell lung cancer	13	51.7 (33.1-70.3)	**IHC evaluation-Percentage**		
Pancreatic cancer	4	47.6 (40.5-54.6)	Cut-off = 5%	19	34.3 (26.5-42.1)
plural mesothelium	1	20.8 (11.7-29.8)	Cut-off = 10%	10	56.5 (49.5-63.6)
Head and neck cancer	4	54.4 (44.8-60.0)	Others	6	42.9 (7.3-78.5)
Skin cancer	5	42.8 (32.5-53.1)	**IHC evaluation-H-score**		
Thymic carcinoma	1	34.5 (26.6-42.4)	Cut-off ≤ 50	10	54.8 (43.1-66.5)
Urothelial carcinoma	6	44.9 (24.4-65.4)	Cut-off > 50	8	37.1 (27.8-46.5)

Abbreviations: CI = confidence interval; IHC = immunohistochemistry; DIA = digital image analysis; NA = not available; PD-L1 = programmed cell death 1ligand 1.

### Correlation of PD-L1 expression and clinicopathological features

#### Age

**Figure 2:**
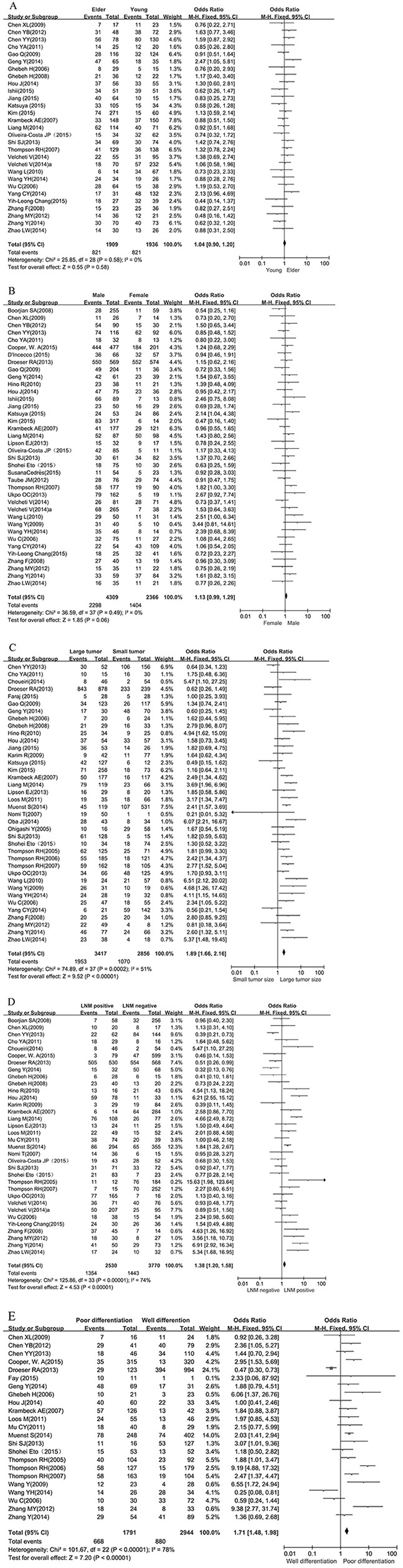
Forest plot for the association between PD-L1 expression and clinicopathological features **A.** age, **B.** gender. Events refer to cases with PD-L1 positive expression. ORs with corresponding 95 % CIs of individual studies and overall are shown in the forest plot. Abbreviations: OR = odds ratio; CI = confidence interval; LNM= lymph node metastasis; PD-L1= Programmed cell death ligand 1. **C.** tumor size, **D.** lymph node metastasis. Events refer to cases with PD-L1 positive expression. ORs with corresponding 95 % CIs of individual studies and overall are shown in the forest plot. Abbreviations: OR = odds ratio; CI = confidence interval; LNM= lymph node metastasis; PD-L1= Programmed cell death ligand 1. **E.** tumor cell differentiation. Events refer to cases with PD-L1 positive expression. ORs with corresponding 95 % CIs of individual studies and overall are shown in the forest plot. Abbreviations: OR = odds ratio; CI = confidence interval; LNM= lymph node metastasis; PD-L1= Programmed cell death ligand 1.

Twenty-nine studies, including 3845 patients, were analyzed for the association between PD-L1 expression and age. Of 1909 elder patients, 821 (43.0%) were PD-L1 expression positive, and 821 (42.4%) of 1936 younger patients were PD-L1 expression positive. No significant association was found between PD-L1 expression and age (OR 1.04, 95% CI 0.90 to 1.20; P = 0.58) (Figure 2A).

#### Gender

Thirty-eight studies, including 6675 patients, were analyzed for the association between PD-L1 expression and gender. Of 4309 male patients, 2298 (53.3%) were PD-L1 expression positive, and 1404 (59.3%) of 2366 female patients were PD-L1 expression positive. No significant association was found between PD-L1 expression and gender (OR 1.13, 95% CI 0.99 to 1.29; P = 0.06) (Figure 2B).

#### Tumor size

The association between PD-L1 expression and size of tumor was analyzed in Thirty-eight studies, including a total of 6273 patients. Of 3417 tumors of large size, 1953 (57.6%) were PD-L1 expression positive, and 1070 (37.5%) of 2856 small size tumors were PD-L1 expression positive. There was a significant association betweenPD-L1 expression and tumors of large size (OR 1.89, 95% CI 1.66 to 2.16; P < 0.01) (Figure 2C).

#### Lymph node metastasis

Thirty-four studies, including 6300 patients, were analyzed for the association between PD-L1 expression and lymph node metastasis. Of 2530 lymph node-positive patients, 1354 (53.5%) tested positive for PD-L1 expression, and 1443 (38.3%) of 3770 lymph node-negative patients tested positive for PD-L1 expression. A significant association was found between PD-L1 expression and the presence of lymph node metastases (OR 1.38, 95% CI 1.20 to 1.58; P < 0.01) (Figure 2D).

#### Differentiation

Twenty-three studies, including 4735 patients, were analyzed for the association of PD-L1 expression and differentiation of tumor cells. Of 1791 patients with poor tumor differentiation (grade III), 668 (37.3%) were PD-L1 expression positive, and 880 (29.9%) of 2944 patients with well differentiation (grade I/II) were PD-L1 expression positive. A significant association exists between PD-L1 expression and tumor cell differentiation (OR 1.71, 95% CI 1.48 to 1.98; P < 0.01) (Figure 2E).

#### Subgroup analysis

Further subgroup analysis was performed by stratifying the studies according to the patients' origins (Asian & Non-Asian), primary anti-PD-L1 antibodies (monoclonal antibody & polyclonal antibody), IHC evaluation for PD-L1 (percentage & H-score), cut-off values for percentage (5% & 10%) and H-score (≤ 50 & > 50). The conclusions drawn from the subgroup analyses are similar to that of the overall analysis (more details are listed in Table 3).

**Table 3:** Summary of subgroup analyses results in studies reported PD-L1 status stratified by clinicopathlogical features

Subgroup	No. of studies; OR [95% CI]				
	Age	Gender	Tumor size	Differentiation	LNM
**Patients' origin**					
Asian	24; 1.03 [0.87, 1.22]	26; 1.19 [1.01, 1.41]	27; 1.75 [1.48, 2.08]	14; 1.58 [1.24, 2.02]	20; 1.53 [1.26, 1.87]
Non-Asian	5; 1.07 [0.82, 1.41]	12; 1.05 [0.85, 1.29]	11; 2.13 [1.73, 2.62]	9; 1.78 [1.49, 2.14]	14; 1.24 [1.02, 1.50]
**Primary anti-PD-L1 antibodies**					
Monoclonal antibody	15; 1.04 [0.86, 1.26]	21; 1.11 [0.94, 1.32]	24; 1.85 [1.57, 2.19]	13; 1.62 [1.34, 1.96]	23; 1.16 [0.96, 1.41]
Polyclonal antibody	13; 1.08 [0.86, 1.34]	16; 1.18 [0.96, 1.45]	13; 2.00 [1.61, 2.48]	9; 1.73 [1.37, 2.18]	11; 1.68 [1.37, 2.06]
**IHC evaluation**					
Percentage	12; 0.98 [0.76, 1.26]	18; 1.16 [0.95, 1.41]	22; 2.16 [1.79, 2.61]	12; 2.61 [2.07, 3.31]	16; 1.61 [1.23, 2.10]
H-score	11; 1.09 [0.88, 1.34]	14; 1.14 [0.93, 1.39]	14; 1.58 [1.30, 1.91]	10; 1.32 [1.09, 1.61]	12; 1.37 [1.13, 1.65]
**IHC evaluation-Percentage**					
Cut-off = 5%	7; 0.97 [0.70, 1.34]	10; 1.17 [0.92, 1.48]	13; 2.15 [1.71, 2.71]	5; 3.50 [2.45, 4.98]	11; 1.82 [1.31, 2.53]
Cut-off = 10%	5; 0.99 [0.67, 1.46]	6; 1.12 [0.71, 1.77]	10; 2.31 [1.66, 3.20]	6; 1.84 [1.28, 2.64]	4; 1.66 [1.00, 2.78]
**IHC evaluation-H-score**					
Cut-off ≤ 50	7; 1.20 [0.92, 1.57]	8; 1.17 [0.90, 1.52]	6; 1.30 [0.96, 1.76]	3; 1.82 [1.16, 2.87]	4; 1.20 [0.86, 1.69]
Cut-off > 50	2; 0.94 [0.59, 1.49]	5; 1.12 [0.81, 1.54]	7; 1.81 [1.41, 2.33]	5; 1.14 [0.91, 1.43]	6; 1.50 [1.17, 1.91]

Abbreviations: CI = confidence interval; OR = Odds ratio; IHC = immunohistochemistry; LNM = lymph node metastasis; PD-L1 = programmed cell death 1ligand 1.

## DISCUSSION

For patients with epithelial-originated malignancies, the association between clinicopathological features and PD-L1 expression remains unclear. A meta-analysis incorporating all available data from correlative studies is a reasonable method to clarify this issue. Through the completion of this meta-analysis we found that epithelial-originated cancer with regional lymph node metastasis, large size tumors, or well differentiated tumors were associated with higher PD-L1 expression rate. We also found that polyclonal antibodies detected a numerically higher PD-L1 expression rate than monoclonal antibodies. Additionally, PD-L1 expression rates reported in studies from Asian areas were numerically higher than those from non-Asian areas namely Caucasians.

We might infer the following as the basis for the positive correlation of PD-L1 expression with large tumor size and lymph node metastasis: under normal conditions, the genetic and epigenetic alterations can distinguish the cancer cells from the normal counterparts, allowing tumors to be recognized and repelled as foreign by the immune system [19]. When PD-L1 is highly expressed on tumor cells, its engagement with PD-1, an important co-inhibitor on the T cell, can block the T cell cytotoxicity and lead to T-cell exhaustion[9]. Binding of PD-L1 to PD-1 on naive tumor-infiltrating lymphocytes (TILs) can arrest the process of antigen presenting at the effector phase and suppress T-cell activation, migration, proliferation and secretion of cytotoxic mediators. Interaction of PD-L1 with PD-1 can also increase apoptosis of tumor specific CD8+ cells [20]. What's more, PD-L1 acts not only as a ligand of PD-1, but can also serve as a receptor transmitting reverse signals that protect cancers cells from apoptosis mediated by FAS-FASL pathway [21]. These actions of PD-L1 expressed on the tumor can thereby dampen the antitumor immunity and contribute the over growth and metastasis of the tumor cells.

The positive correlation between PD-L1 expression and poor differentiation of tumors found in this meta-analysis is consistent with the majority of other studies, though the underlying mechanism remains unclear. Poor differentiation may be associated with over proliferating effect of the PD-L1 positive tumor cells. Quite a lot published evidences have shown that PD-L1 expression promote tumor cell proliferation indirectly through both exogenous and intrinsic pathway. On one hand, exogenous stimulus delivered by the pro-inflammatory cytokines in the inflammatory microenvironment can trigger the receptor mediated signaling molecules within the tumor cells (including NF-κB, MAPK, PI3K, mTOR, and JAK/STAT) that promote cell proliferation, and induce PD-L1 expression as well [22]. On the other hand, some intrinsic oncogenic pathway (like anaplastic lymphoma kinase in lung cancer [23], loss of tumor suppressor phosphate and tension homolog in pancreatic cancer [24] etc.) have been reported to drive PD-L1 expression and, in the meanwhile, are involved in key cellular functions such as proliferation, growth, and survival [25–27]. Over proliferation of the tumor cells is usually accompanied with cellular de-differentiation, which explains the positive correlation between poor differentiation and PD-L1 over expression of the tumor cells.

With regard to the immune evasion of solid tumor mediated by PD-1/PD-L1 immune-checkpoint pathway, a series of specific inhibitors are currently under investigation and in clinical development such as nivolumab, a fully human IgG4 PD-1 immune checkpoint inhibitor antibody, pembrolizumab (formerly known as MK-3475 or lambrolizumab), a high affinity humanized IgG4 monoclonal antibody targeting PD-1, and MPDL3280A, an engineered IgG anti-PD-L1 antibody [11, 12, 15, 28]. Among these, pembrolimumab and nivolumab [29, 30] have been approved by the US Food and Drug administration (FDA) in the treatment of advanced melanoma and non-small cell lung cancer for their remarkably durable clinical response. Along with that, two different immunohistochemical (IHC) assays that linked to the use of pembrolizumab and nivolumab will come into the market for PD-L1 expression detection[31]. Although the therapeutic effect of anti-PD-1/PD-L1 treatment is unprecedented, the response rate in the context of unselected population affected by advanced solid tumors was barely satisfactory, ranging from 10% to 45% [32–34]. In light of the expense and adverse effects of anti-PD-1/PD-L1 therapy, further exploration is warranted to identify the proportion of patients most likely to benefit from the immunotherapy and thus optimize their therapeutic index. More and more evidences indicated that PD-L1 expression level in tumor cells is positively correlated with the response rate [17, 35]. It's well known that epidermal growth factor receptor gene (*EGFR*) mutation status has a similar predictive role in the efficacy of EGFR-targeting therapy for patients with non-small cell lung cancer (NSCLC) [34]. But what differs PD-L1 from the EGFR protein is the lack of a clear-cut gene-mediated pathway that exclusively contributes to the over expression of PD-L1. That means we can't precisely identify PD-L1 status at the gene level as multiple (intrinsic and extrinsic) signaling pathways are involved in the induction of PD-L1 expression [37]. As for the quantitative or semi-quantitative detection of PD-L1 by immunohistochemistry (IHC), its reliance and reproducibility is still disputed due to the lack of uniform antibody and cut-off values, which consequently restricts its application in screening candidate patients for the immunotherapy. What's more, PD-L1 expression may change dynamically during the process of tumor progression. It's unreliable to decide PD-L1 status by IHC detection at a single time point. Considering the dilemma of PD-L1 detection in clinical practice, a more practical way of patients' stratification is warranted. The elucidation of the relationship between PD-L1 expression and clinicopathological parameters in this meta-analysis provides a convenient way to identify patients that are most likely to have a high level of PD-L1 expression on the tumor cells and thus provides rationale for patients' stratification in clinical practice. According to our pooled analysis, patients with larger tumor size, lymph node metastasis, or poorly differentiated tumors tend to have higher level of PD-L1 expression; these patients may benefit more from treatment targeting PD-1/PD-L1 pathway. What's more, as these clinicopathological parameters are also associated with advanced stage and poor prognosis, our results rationalize the application of anti-PD-1/PD-L1 immunotherapy in patients with advanced tumors that currently lack effective treatment options. It also indirectly proves that PD-L1 over expression is associated with poor prognosis of solid tumors as demonstrated by previous studies [38].

The higher expression rate of PD-L1 among patients of Asian origin, as shown in our results, may be associated with higher frequencies of virus-associated malignancies in Asian areas. PD-L1 over expression has been reported to be associated with viral infection and chronic inflammation [39]. Intratumoral expression of PD-L1 and/or PD-1 has been shown in polyomavirus-associated Merkel cell carcinoma [40], hepatitis B virus(HBV)-related hepatocellular carcinoma [41], human papillomavirus(HPV)-associated head and neck cancer [42], Epstein Barr Virus(EBV)-associated nasopharyngeal cancer [43] and so on. Our result implies that the immune associated PD-1/PD-L1 may play a significant role in the development and progression of virus associated cancer.

To the best of our knowledge, this is the first study to comprehensively demonstrate the relationship between PD-L1 status and clinicopathological parameters of solid tumors. However, several limitations existed: (I) Different tumors may have different biologic behaviors, but we failed to conduct this particular subgroup analysis as data for some tumor types was insufficient; (II) Cut-off values distinguishing high or low levels of PD-L1 expression determined by IHC evaluation and the primary antibodies varied in different types of tumors, which might cause heterogeneity of the overall results; (III) Different studies adopt different thresholds for the size of large tumors, as well as the age for elder patients, which can also lead to the heterogeneity of the pooled result. (IV) As a result of the lack of sufficient data, we were unable to evaluate the relationship between clinicopathological parameters and PD-1/PD-L1 status on the tumor infiltrating lymphocytes (TILs) which is also associated with immune evasion of tumor and responsiveness of immunotherapy.

Regardless of the above limitations, this comprehensive analysis demonstrates the relationship between PD-L1 expression and clinicopathological features of solid tumors. The results may lead to improvement in the outcome of anti-PD-1/PD-L1 therapy by guiding patients' stratification in a convenient way. Further large-scale clinical studies should be performed to demonstrate the association between PD-L1 expression and clinicopathological characteristics in the subgroups of different tumor types. Further effort is also warranted to investigate the relationship between PD-1/PD-L1 status on TILs.

In summary, meta-analysis of the literature shows that PD-L1 over expression on the tumor cells correlates with poor prognostic features including large tumor size, lymph node metastasis and poor differentiation of solid tumors. The PD-L1 expression rate in Asians might be higher than that in Caucasians. This information might prove to be helpful in screening candidates for relevant treatments.

## MATERIALS AND METHODS

### Search strategy

We conducted a systematic search of PubMed, Embase, and Cochrane databases for articles published from inception to July 2015. The following keywords were used: “PD-L1” or “B7-H1” or “CD274” or “PD-1” or “CD279” or “programmed cell death 1” combined with “cancer” or “tumor” or “carcinoma” with limits “human”. An additional search through Google Scholar and a manual search through reference lists of relevant reviews were additionally performed. Three authors (ZY, KS and SJ) independently carried out the search. As Chinese investigators, we restricted our searches to studies published in either English or Chinese.

### Inclusion and exclusion criteria

Eligible studies fulfilled the following inclusion criteria: (i) original articles; (ii) the expression level of PD-L1 is tested by immunohistochemistry (IHC) staining on tumor cell specimens; (iii) data of the binary clinicopathological factors stratified by PD-L1 status were available. Studies that failed to meet the inclusion criteria were excluded.

### Data extraction

The data collection and assessment of methodological quality followed the QUORUM and the Cochrane Collaboration guidelines (http://www.cochrane.de). The data on lead author, publication year, tumor type, patients' characteristics, patient origin, primary antibody, and PD-L1 status, clinicopathological features stratified by PD-L1 status were extracted by two investigators (LZY and XYT) independently. For uniformed data analysis, T1 was considered as small size, and T2, T3, and T4 as large size for CRC, EC and UC. For the histological grade, grades I and II (well/moderate differentiation) were grouped together *vs* grade III (poor differentiation). We defined large tumors in other tumor types and elder patients according to the criteria of each individual study. Reviewers (LZY and XYT) used the Newcastle-Ottawa scale specific to cohort study to assess all included studies. Discrepancies were discussed by all investigators to reach consensus. All eligible studies were of high quality.

### Statistical analysis

Analysis was performed using Stata 12.0 (Stata Corporation, Texas, US) and Review Manager 5.2 (Cochrane Collaboration, Oxford, UK). The relationship between PD-L1 expression rate and antibody types, patients' origin, and IHC evaluation methods were investigated using independent statistical t-test respectively. Comparisons of dichotomous measures were performed by pooled estimates of odds ratios (ORs), as well as their 95% CI. Subgroup analysis was conducted according to patients' origin, primary antibody (monoclonal antibody & polyclonal antibody) and IHC evaluation method (even in different cut-off values for PD-L1 positive) respectively. All CIs had two-sided probability coverage of 95%. A statistical test with P-value less than 0.05 was considered significant.

### Publication bias

An extensive search strategy was made to minimize the potential publication bias. Graphical funnel plots were generated to visually assess publication bias. The statistical method to detect funnel plot asymmetry was the Begg's test.

## SUPPLEMENTARY FIGURE



## References

[1] Greenwald RJ, Freeman GJ, Sharpe AH (2005). The B7 family revisited. Annual review of immunology.

[2] Jin HT, Ahmed R, Okazaki T (2011). Role of PD-1 in regulating T-cell immunity. Current topics in microbiology and immunology.

[3] Zha Y, Blank C, Gajewski TF (2004). Negative regulation of T-cell function by PD-1. Critical reviews in immunology.

[4] D'Incecco A, Andreozzi M, Ludovini V, Rossi E, Capodanno A, Landi L, Tibaldi C, Minuti G, Salvini J, Coppi E, Chella A, Fontanini G, Filice ME, Tornillo L, Incensati RM, Sani S (2015). PD-1 and PD-L1 expression in molecularly selected non-small-cell lung cancer patients. British journal of cancer.

[5] Faraj SF, Munari E, Guner G, Taube J, Anders R, Hicks J, Meeker A, Schoenberg M, Bivalacqua T, Drake C, Netto GJ (2015). Assessment of tumoral PD-L1 expression and intratumoral CD8+ T cells in urothelial carcinoma. Urology.

[6] Yamane H, Isozaki H, Takeyama M, Ochi N, Kudo K, Honda Y, Yamagishi T, Kubo T, Kiura K, Takigawa N (2015). Programmed cell death protein 1 and programmed death-ligand 1 are expressed on the surface of some small-cell lung cancer lines. American journal of cancer research.

[7] Mazel M, Jacot W, Pantel K, Bartkowiak K, Topart D, Cayrefourcq L, Rossille D, Maudelonde T, Fest T, Alix-Panabieres C (2015). Frequent expression of PD-L1 on circulating breast cancer cells. Mol Oncol.

[8] Chang YL, Yang CY, Lin MW, Wu CT, Yang PC (2015). PD-L1 is highly expressed in lung lymphoepithelioma-like carcinoma: A potential rationale for immunotherapy. Lung cancer.

[9] Topalian SL, Drake CG, Pardoll DM (2012). Targeting the PD-1/B7-H1(PD-L1) pathway to activate anti-tumor immunity. Current opinion in immunology.

[10] Flies DB, Sandler BJ, Sznol M, Chen L (2011). Blockade of the B7-H1/PD-1 pathway for cancer immunotherapy. The Yale journal of biology and medicine.

[11] Tsai KK, Zarzoso I, Daud AI (2014). PD-1 and PD-L1 antibodies for melanoma. Human vaccines & immunotherapeutics.

[12] Brower V (2015). Anti-PD-L1 antibody active in metastatic bladder cancer. The Lancet Oncology.

[13] Gettinger S, Herbst RS (2014). B7-H1/PD-1 blockade therapy in non-small cell lung cancer: current status and future direction. Cancer journal.

[14] Massari F, Santoni M, Ciccarese C, Santini D, Alfieri S, Martignoni G, Brunelli M, Piva F, Berardi R, Montironi R, Porta C, Cascinu S, Tortora G (2015). PD-1 blockade therapy in renal cell carcinoma: Current studies and future promises. Cancer treatment reviews.

[15] Ohaegbulam KC, Assal A, Lazar-Molnar E, Yao Y, Zang X (2015). Human cancer immunotherapy with antibodies to the PD-1 and PD-L1 pathway. Trends in molecular medicine.

[16] Errico A (2015). Immunotherapy: PD-1-PD-L1 axis: efficient checkpoint blockade against cancer. Nature reviews Clinical oncology.

[17] Patel SP, Kurzrock R (2015). PD-L1 Expression as a Predictive Biomarker in Cancer Immunotherapy. Mol Cancer Ther.

[18] Carbognin L, Pilotto S, Milella M, Vaccaro V, Brunelli M, Calio A, Cuppone F, Sperduti I, Giannarelli D, Chilosi M, Bronte V, Scarpa A, Bria E, Tortora G (2015). Differential Activity of Nivolumab, Pembrolizumab and MPDL3280A according to the Tumor Expression of Programmed Death-Ligand-1 (PD-L1): Sensitivity Analysis of Trials in Melanoma, Lung and Genitourinary Cancers. PloS one.

[19] Postow MA, Callahan MK, Wolchok JD (2015). Immune Checkpoint Blockade in Cancer Therapy. Journal of clinical oncology.

[20] Pauken KE, Wherry EJ (2015). Overcoming T cell exhaustion in infection and cancer. Trends in immunology.

[21] Sanmamed MF, Chen L (2014). Inducible expression of B7-H1 (PD-L1) and its selective role in tumor site immune modulation. Cancer journal.

[22] Ritprajak P, Azuma M (2015). Intrinsic and extrinsic control of expression of the immunoregulatory molecule PD-L1 in epithelial cells and squamous cell carcinoma. Oral oncology.

[23] Wasik MA, Zhang Q, Marzec M, Kasprzycka M, Wang HY, Liu X (2009). Anaplastic lymphoma kinase (ALK)-induced malignancies: novel mechanisms of cell transformation and potential therapeutic approaches. Seminars in oncology.

[24] Zhang Y, Zhang J, Xu K, Xiao Z, Sun J, Xu J, Wang J, Tang Q (2013). PTEN/PI3K/mTOR/B7-H1 signaling pathway regulates cell progression and immuno-resistance in pancreatic cancer. Hepato-gastroenterology.

[25] Abildgaard C, Guldberg P (2015). Molecular drivers of cellular metabolic reprogramming in melanoma. Trends in molecular medicine.

[26] Kalia M (2015). Biomarkers for personalized oncology: recent advances and future challenges. Metabolism: clinical and experimental.

[27] Milella M, Falcone I, Conciatori F, Cesta Incani U, Del Curatolo A, Inzerilli N, Nuzzo CM, Vaccaro V, Vari S, Cognetti F, Ciuffreda L (2015). PTEN: Multiple Functions in Human Malignant Tumors. Frontiers in oncology.

[28] Barbee MS, Ogunniyi A, Horvat TZ, Dang TO (2015). Current Status and Future Directions of the Immune Checkpoint Inhibitors Ipilimumab, Pembrolizumab, and Nivolumab in Oncology. The Annals of pharmacotherapy.

[29] McDermott J, Jimeno A (2015). Pembrolizumab: PD-1 inhibition as a therapeutic strategy in cancer. Drugs of today (Barc).

[30] Gunturi A, McDermott DF (2015). Nivolumab for the treatment of cancer. Expert opinion on investigational drugs.

[31] Jorgensen JT (2015). Companion diagnostic assays for PD-1/PD-L1 checkpoint inhibitors in NSCLC. Expert review of molecular diagnostics.

[32] Freeman-Keller M, Weber JS (2015). Anti-programmed death receptor 1 immunotherapy in melanoma: rationale, evidence and clinical potential. Therapeutic advances in medical oncology.

[33] Marquez-Rodas I, Cerezuela P, Soria A, Berrocal A, Riso A, Gonzalez-Cao M, Martin-Algarra S (2015). Immune checkpoint inhibitors: therapeutic advances in melanoma. Annals of translational medicine.

[34] Jia M, Feng W, Kang S, Zhang Y, Shen J, He J, Jiang L, Wang W, Guo Z, Peng G, Chen G, He J, Liang W (2015). Evaluation of the efficacy and safety of anti-PD-1 and anti-PD-L1 antibody in the treatment of non-small cell lung cancer (NSCLC): a meta-analysis. Journal of thoracic disease.

[35] Herbst RS, Soria JC, Kowanetz M, Fine GD, Hamid O, Gordon MS, Sosman JA, McDermott DF, Powderly JD, Gettinger SN, Kohrt HE, Horn L, Lawrence DP, Rost S, Leabman M, Xiao Y (2014). Predictive correlates of response to the anti-PD-L1 antibody MPDL3280A in cancer patients. Nature.

[36] Popper HH, Ryska A, Timar J, Olszewski W (2014). Molecular testing in lung cancer in the era of precision medicine. Translational lung cancer research.

[37] Van Allen EM, Golay HG, Liu Y, Koyama S, Wong K, Taylor-Weiner A, Giannakis M, Harden M, Rojas-Rudilla V, Chevalier A, Thai T, Lydon C, Mach S, Avila AG, Wong JA, Rabin AR (2015). Long-term Benefit of PD-L1 Blockade in Lung Cancer Associated with JAK3 Activation. Cancer Immunol Res.

[38] Wu P, Wu D, Li L, Chai Y, Huang J (2015). PD-L1 and Survival in Solid Tumors: A Meta-Analysis. PloS one.

[39] Abdel-Magid AF (2015). Inhibitors of the PD-1/PD-L1 Pathway Can Mobilize the Immune System: An Innovative Potential Therapy for Cancer and Chronic Infections. ACS medicinal chemistry letters.

[40] Lipson EJ, Vincent JG, Loyo M, Kagohara LT, Luber BS, Wang H, Xu H, Nayar SK, Wang TS, Sidransky D, Anders RA, Topalian SL, Taube JM (2013). PD-L1 expression in the Merkel cell carcinoma microenvironment: association with inflammation, Merkel cell polyomavirus and overall survival. Cancer immunology research.

[41] Zeng Z, Shi F, Zhou L, Zhang MN, Chen Y, Chang XJ, Lu YY, Bai WL, Qu JH, Wang CP, Wang H, Lou M, Wang FS, Lv JY, Yang YP (2011). Upregulation of circulating PD-L1/PD-1 is associated with poor post-cryoablation prognosis in patients with HBV-related hepatocellular carcinoma. PloS one.

[42] Lyford-Pike S, Peng S, Young GD, Taube JM, Westra WH, Akpeng B, Bruno TC, Richmon JD, Wang H, Bishop JA, Chen L, Drake CG, Topalian SL, Pardoll DM, Pai SI (2013). Evidence for a role of the PD-1:PD-L1 pathway in immune resistance of HPV-associated head and neck squamous cell carcinoma. Cancer research.

[43] Fang W, Zhang J, Hong S, Zhan J, Chen N, Qin T, Tang Y, Zhang Y, Kang S, Zhou T, Wu X, Liang W, Hu Z, Ma Y, Zhao Y, Tian Y (2014). EBV-driven LMP1 and IFN-gamma up-regulate PD-L1 in nasopharyngeal carcinoma: Implications for oncotargeted therapy. Oncotarget.

