# Supplementary material for: The prevalence and clinicopathological features of programmed death-ligand 1 (PD-L1) expression: a pooled analysis of literatures
Source: Oncotarget. 2016 Feb 22;7(12):15033–46. doi: 10.18632/oncotarget.7590 (PMC4924769; doi:10.18632/oncotarget.7590)
Supplement: Supplementary file 1 [file oncotarget-07-15033-s001.pdf]

SUPPLEMENTARY FIGURE

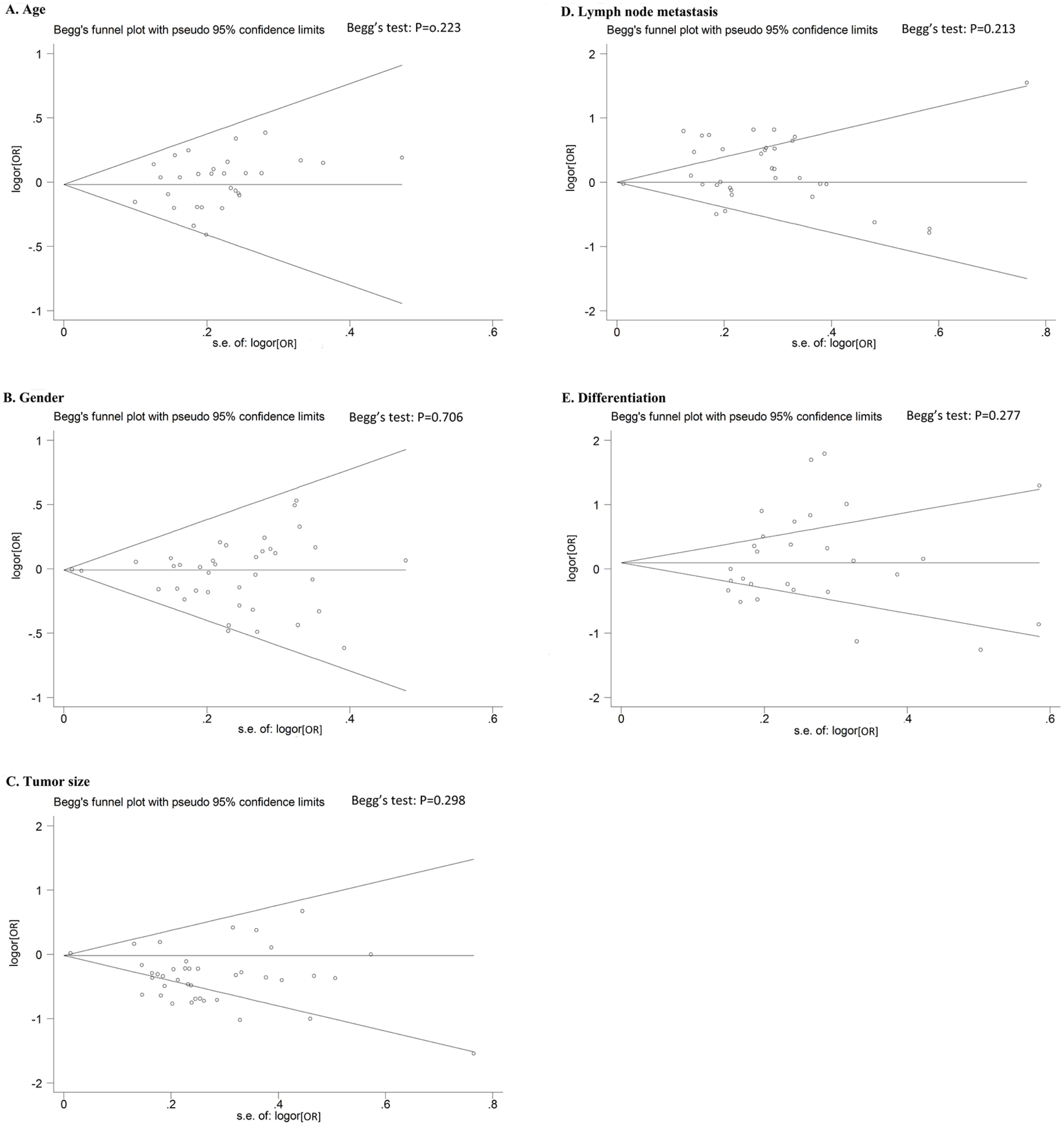

Supplementary Figure S1: Graphical funnel plot with the Begg's test of the overall result. A. age, **B.** gender, **C.** tumor size, **D.** lymph node metastasis, **E.** tumor cell differentiation. Abbreviations: OR = odds ratio; PD-L1 = programmed cell death 1 ligand 1.
